# Supplementary material for: Novel Energetic Co-Reactant for Thermal Oxide Atomic Layer Deposition: The Impact of Plasma-Activated Water on Al2O3 Film Growth
Source: Nanomaterials (Basel). 2023 Dec 10;13(24):3110. doi: 10.3390/nano13243110 (PMC10745439; doi:10.3390/nano13243110)
Supplement: Supplementary file 1 [file nanomaterials-13-03110-s001.zip › nanomaterials-2743970-supplementary.pdf]

Supporting Information

Novel Energetic Co-Reactant for Thermal ALD: The Impact of Plasma-Activated Water on Al_2O_3 Film Growth

João Chaves [1,†], William Chiappim [2], Júlia Karnopp [1], Benedito Neto [1], Douglas Leite [1], Argemiro da Silva Sobrinho [1] and Rodrigo Pessoa [1,*]

[1] Laboratório de Plasmas e Processos, Departamento de Física, Instituto Tecnológico de Aeronáutica, Praça Marechal Eduardo Gomes 50, São José dos Campos 12228-900, Brazil; julia_karnopp@outlook.com (J.K.); botan.bdn@gmail.com (B.N.); leite@ita.br (D.L.); argemiro@ita.br (A.d.S.S.)

[2] Laboratório de Plasmas e Aplicações, Departamento de Física, Faculdade de Engenharia de Guaratinguetá, São Paulo State University (UNESP), Guaratinguetá 12516-410, Brazil; chiappimjr@yahoo.com.br

* Correspondence: rspessoa@ita.br; Tel.: +55-12-3947-5785

† In memoriam.

1. The experimental setup for PAW generation and the electrical characterization of the gliding arc plasma jet

The experimental setup for plasma-activated water (PAW) generation consists of a plasma reactor, a high-voltage power supply, an oscilloscope, and a substrate holder (Fig. S1). A gliding arc plasma jet (GAPJ) is generated in a Forward Vortex Flow Reactor (FVFR) configuration [24], with its outer region comprising the plasma plume and post-discharge areas [24,25]. In this study, the gas employed is air, produced by an air compressor (Schulz CSD 9/50, Joinville, SC, Brazil) at a flow rate of 5 L min^{-1}. This airflow was selected to facilitate the formation of a continuous gliding arc discharge with the lowest possible flow, as demonstrated by Doria et al. [24]. The system is powered by a high-voltage power supply (Arternis 0215 model, Inergiae, Florianópolis, SC, Brazil), operating at a frequency of 20 kHz.

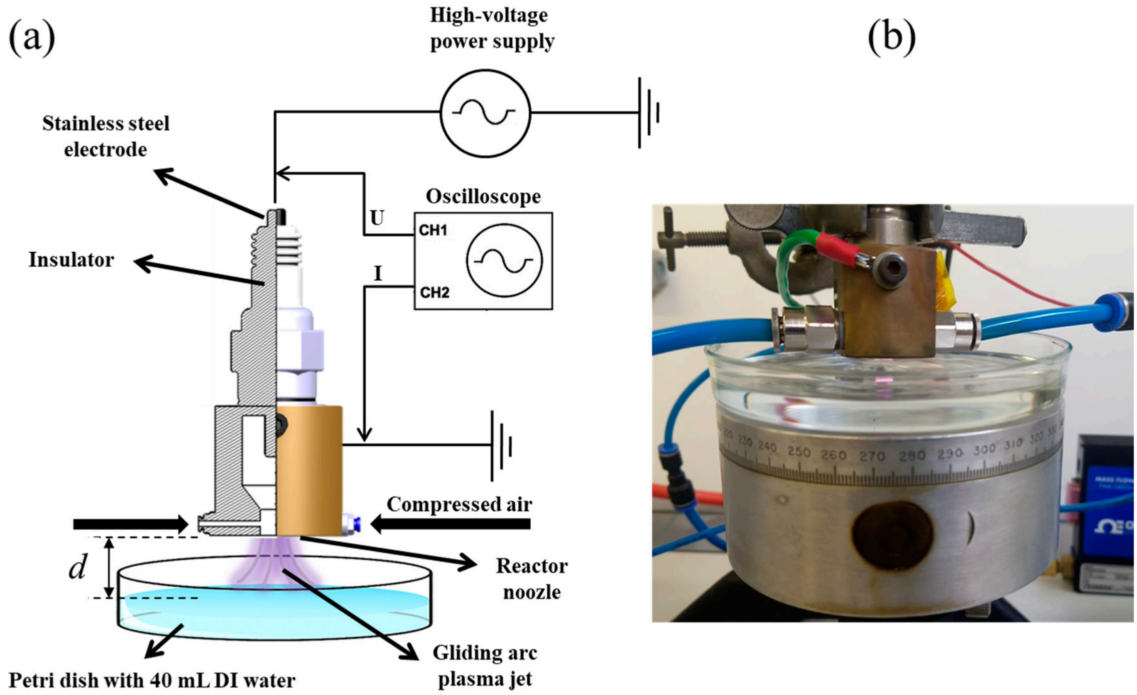

Figure S1. (a) Schematic illustration of the experimental setup, and (b) photograph of the FVFR gliding arc reactor used for activating deionized (DI) water. The value of d was fixed in 3 mm.

The voltage and current waveforms of the GAPJ discharge (Fig. S2) were acquired using a high-voltage probe (Tektronix P6015A, Tektronix, Beaverton, OR, USA) and a self-adjusting current probe (Agilent N2869B, Agilent, Santa Clara, CA, USA). Electrical signals were captured by a digital oscilloscope (Keysight DSOX1202A, Keysight, Santa Rosa, CA, USA), with the current signal being directly inferred from the grounded electrode. Figures S2a and S2b display the voltage and current characteristic curves as functions of time, respectively. The equation (S1) and Fig. S2c were utilized to calculate the power dissipated in the plasma [24].

$$P_{dissip}(W) = \frac{1}{T_2 - T_1}\int_{T_1}^{T_2} V(t)I(t)dt \qquad \text{(S1)}$$

where $V(t)$ is the voltage, $I(t)$ is the electric current and $T_2 - T_1$ is the time interval.

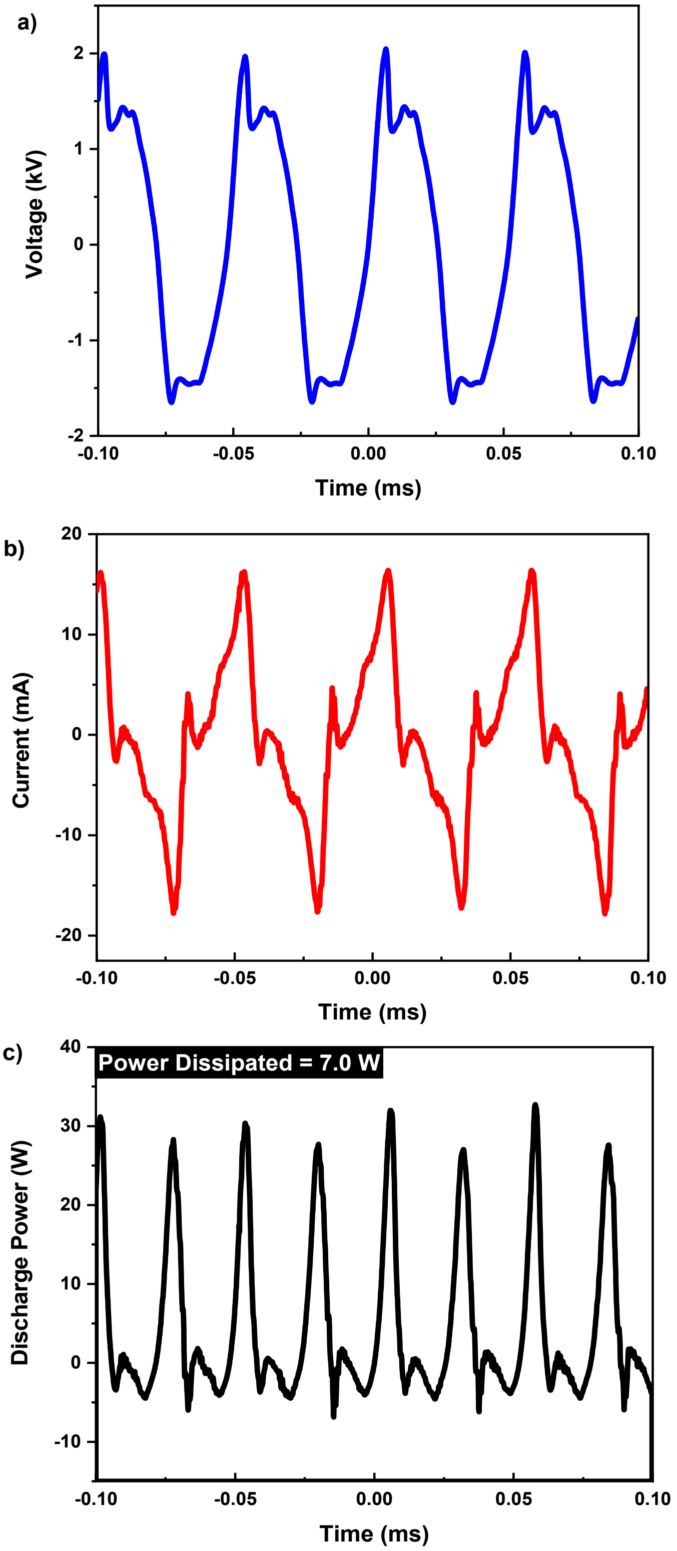

Figure S2. (a) Voltage waveform for the GAPJ operating with an airflow of 5 L min^{-1}, (b) Current waveform for the GAPJ operating with an airflow of 5 L min^{-1}, and (c) Discharge power waveform of the GAPJ used to calculate the mean power using Equation (S1).

The obtained electrical parameters were a peak-to-peak voltage of 3.6 kV, a peak-to-peak current of 33 mA, and a dissipated power of 7 W.

2. UV-Vis spectroscopy of plasma-activated water

A UV-Vis spectrophotometer (Evolution 201/220, Thermo Fisher Scientific, Waltham, MA, USA) was utilized to detect the presence of reactive oxygen and nitrogen species (RONS) in *ex-situ* plasma activated water (PAW) determined by measuring optical absorbance in the UV-Vis region (190–900 nm). The UV-Vis spectrophotometer was configured with a spectral resolution of 0.2 nm and a scan speed of 120 nm/min. The samples were placed in a 3.5 mL square quartz cuvette featuring a standard optical path of 10 mm with two polished sides.

A background curve was obtained using an empty quartz cuvette to acquire the UV-Vis spectrum. Subsequently, a quartz cuvette containing 3.5 mL of deionized water (pH = 6.7) was employed to establish the baseline. This approach ensured that the intensities observed in the UV-Vis spectrum of the PAW were attributable to the RONS. After obtaining the baseline (DI water spectrum), the cuvettes were cleaned, and aliquots of PAW (pH 2.7, 3.1, or 3.5) were added. The relative UV absorption spectrum of each PAW was then acquired according to reference [10,26,27] (see Fig. S3).

Figures S3a, S3b, and S3c present the UV-Vis spectra in the deep UV region. The deconvolution curves used to identify long-lived RONS, specifically hydrogen peroxide (H_2O_2), nitrous acid (HNO_2), nitrite (NO_2^-), nitrate (NO_3^-), and ozone (O_3), are also illustrated. Gaussian functions were employed to obtain better fitting deconvolution curves representing the RONS. All curves exhibit a baseline and an envelope. The primary objective is to locate the RONS peaks using Gaussian functions, and ultimately, the sum of all Gaussian functions representing the respective RONS should generate an envelope curve that fits the experimental data.

As observed in the spectra of Fig. S3b and S3c, the same model with six Gaussian functions was applied to determine the individual peaks of contributions from species H_2O_2 (190 nm) [10,26,27]; HNO_2 (195 nm) [28]; NO_3^- (202-205 nm) and (218-222 nm) [10,26,27]; NO_2^- (210-214 nm) [10,26,27] and (230 nm) [10,26,27]; and O_3 (240-260 nm) [28]. However, Fig. S3a representing the spectrum of the PAW with pH 2.7, exhibits a shoulder with a peak at 230 nm, differing from the smoothness of the spectra obtained for pH values of 3.5 and 3.1. Consequently, an additional 7th Gaussian function was required for the envelope to perfectly fit the experimental data. The extra Gaussian function did not alter the results, as it describes a new peak position for NO_2^-, which aligns with the literature [29].

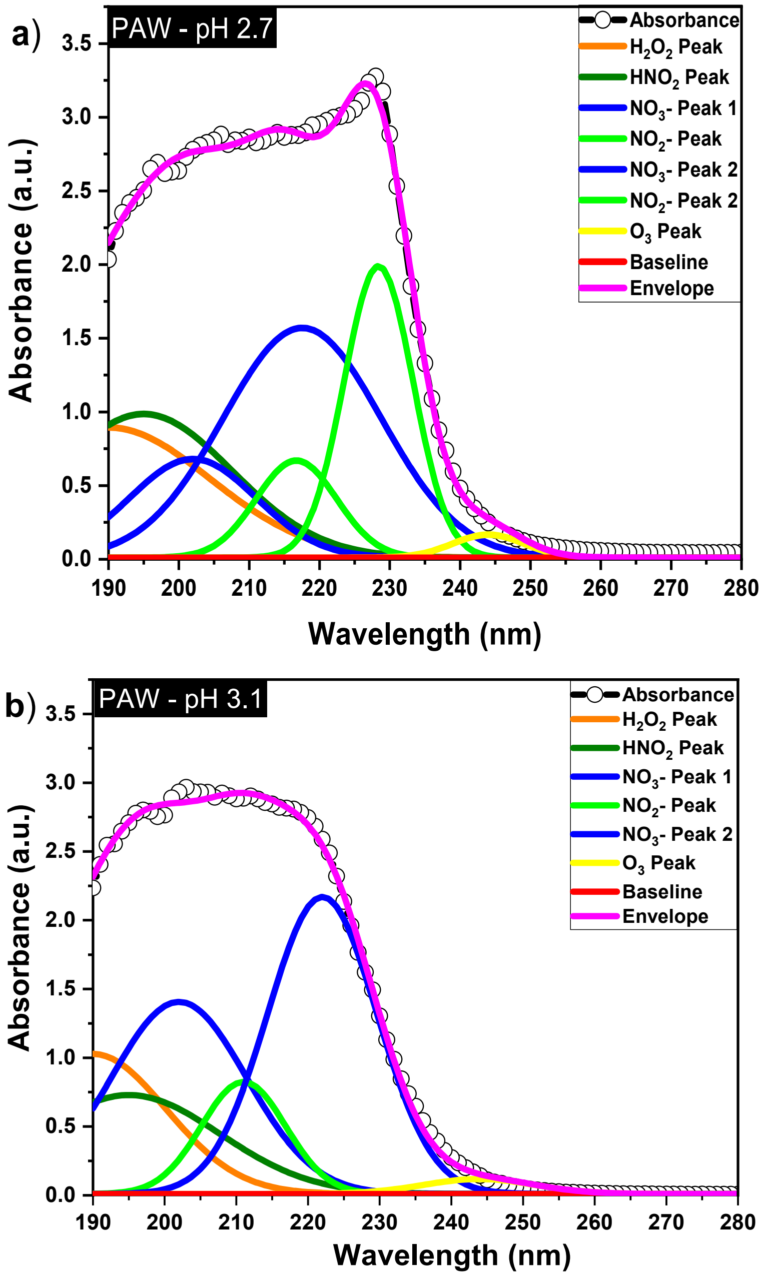
a)
PAW - pH 2.7
Absorbance
H2O2 Peak
HNO2 Peak
NO3- Peak 1
NO2- Peak
NO3- Peak 2
NO2- Peak 2
O3 Peak
Baseline
Envelope
Absorbance (a.u.)
Wavelength (nm)
0.0
0.5
1.0
1.5
2.0
2.5
3.0
3.5
190
200
210
220
230
240
250
260
270
280
b)
PAW - pH 3.1
Absorbance
H2O2 Peak
HNO2 Peak
NO3- Peak 1
NO2- Peak
NO3- Peak 2
O3 Peak
Baseline
Envelope
Absorbance (a.u.)
Wavelength (nm)

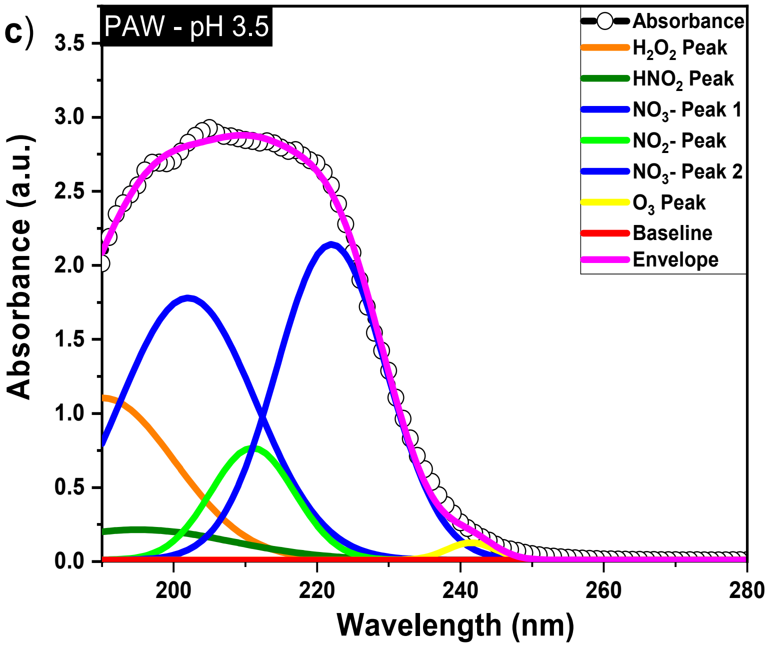

Figure S3. Deep UV absorption spectra of PAW at different pH values: (a) 2.7, (b) 3.1, and (c) 3.5, with non-activated water used as the baseline. Gaussian deconvolution of the deep UV absorption spectrum of each investigated PAW was performed based on literature data [10,26,27].

3. Absolute concentrations of H_2O_2, NO_3^-, NO_2^-, and HNO_2

In the present study, we employ the technique developed by Liu et al. [27] to determine the concentrations of H_2O_2, NO_3^-, and NO_2^- (Table 1 in manuscript). This approach is utilized to minimize significant errors in the calculated RONS concentration, as the absorption lines of H_2O_2, NO_3^-, and NO_2^- overlap between 190 and 230 nm, which can be observed in the deconvolutions presented in Fig. S3 [27]. The UV-Vis spectra depicted in Fig. S3 were employed for this analysis. Utilizing equation (S2), the matrix (S3) was established, and then solved simultaneously to obtain the concentrations of H_2O_2, NO_3^-, and NO_2^- in mg/L.

$$A_\lambda = \alpha[NO_3^-] + \beta[NO_2^-] + \varepsilon[H_2O_2] \quad \text{(S2)}$$

$$\begin{pmatrix} [NO_3^-] \\ [NO_2^-] \\ [H_2O_2] \end{pmatrix} = \begin{pmatrix} \alpha(\lambda_1) & \beta(\lambda_1) & \varepsilon(\lambda_1) \\ \alpha(\lambda_2) & \beta(\lambda_2) & \varepsilon(\lambda_2) \end{pmatrix} \begin{pmatrix} A_{\lambda 1} \\ A_{\lambda 2} \end{pmatrix} / l \quad \text{(S3)}$$

where α, β, and ε are the molar absorptivity coefficients and l denotes the optical path length.

The simultaneous solution was carried out for wavelengths specified at 230 nm, 235 nm, and 250 nm, in accordance with reference [27]. By using the equation (S4), the concentration of nitrous acid (HNO_2) was determined, since, as stated by Tachibana et al. [28], the composition of nitrous acid (HNO_2) and its ion (NO_2^-) depends on the pH level of the solution, with the acidity constant of HNO_2 being $pKa = 3.38$ at room temperature.

$$[HNO_2] = \frac{[NO_2^-]}{10^{pH-pKa}} \tag{S4}$$

4. XPS spectra deconvolution and analysis

To ascertain the chemical composition and binding states of Al_2O_3 films, XPS measurements were conducted. The survey XPS spectra of studied Al_2O_3 films are presented in Fig. S4. The samples were scanned across a binding energy range of 0 to 1250 eV. The survey spectra of Al_2O_3 films reveal the presence of several distinct peaks at binding energies of 74 eV, 118 eV, 285 eV, 531 eV, 975 eV, and 1230 eV. These correspond to the Al 2p, Al 2s, C 1s, O 1s, O KLL, and C KLL, respectively [30,31].

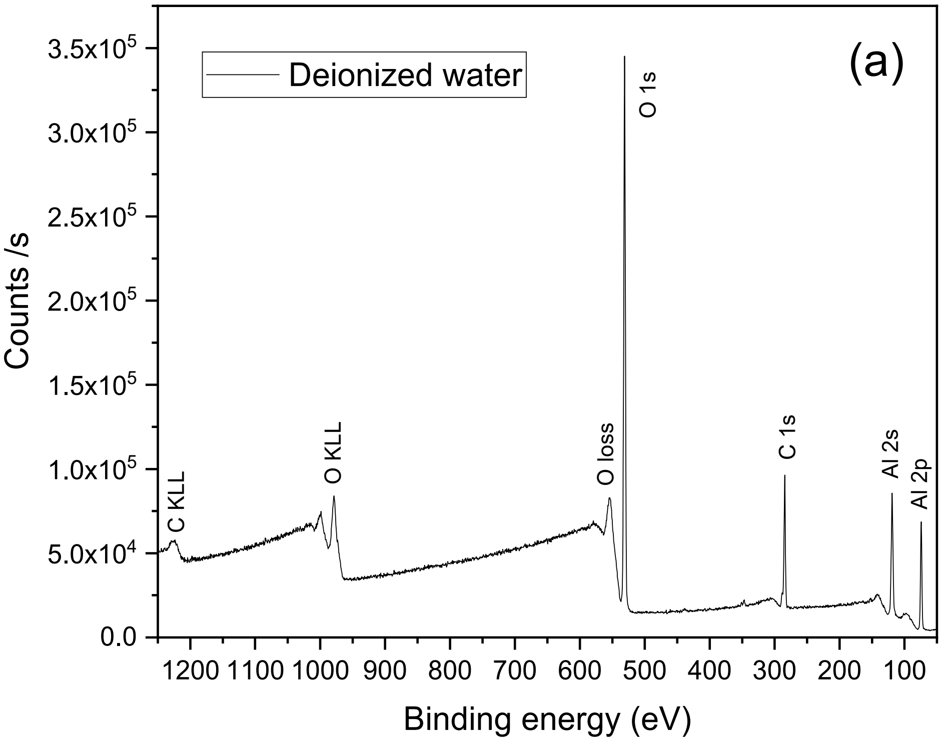

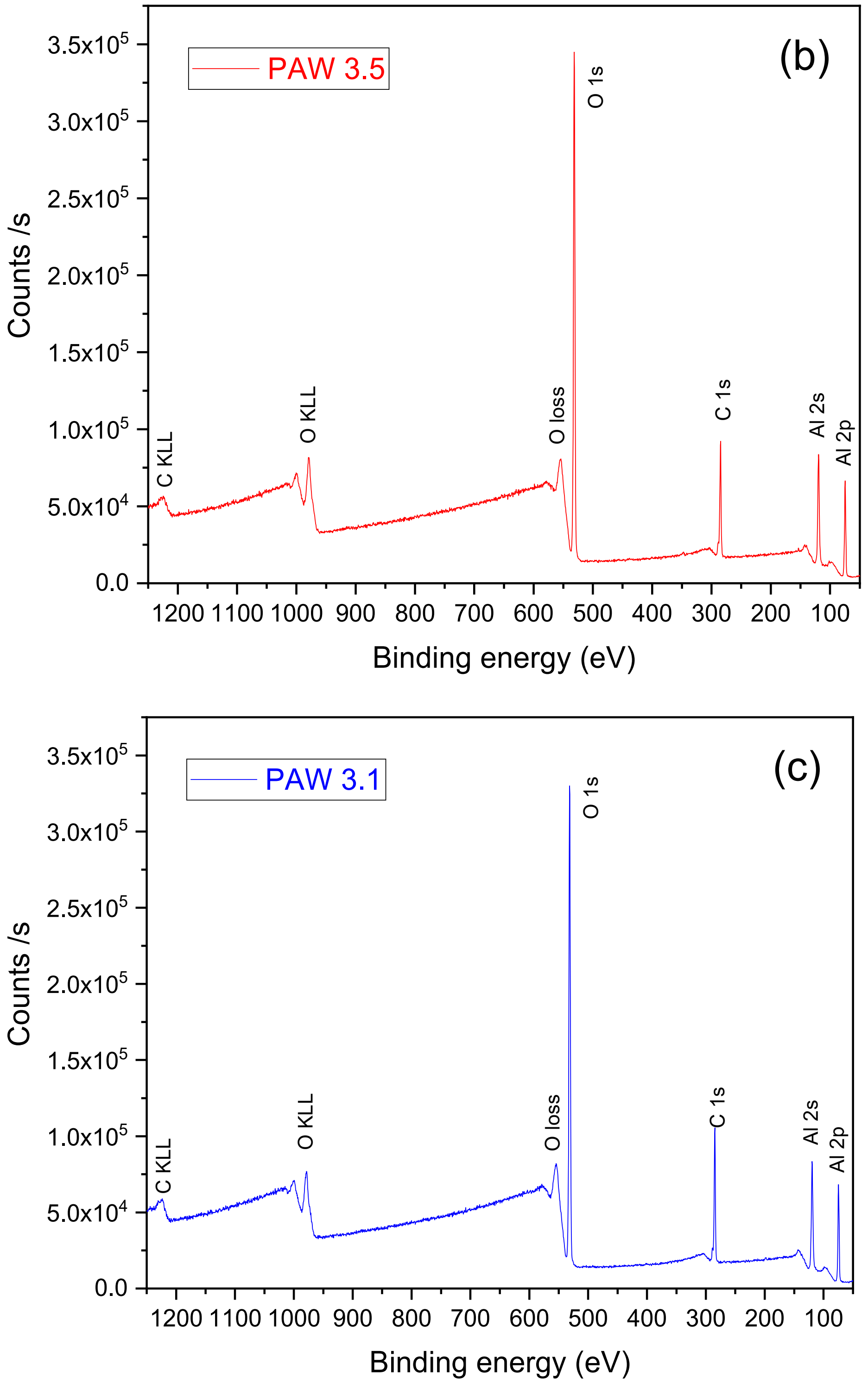
(b)
PAW 3.5
Counts /s
Binding energy (eV)
O 1s
C KLL
O KLL
O loss
C 1s
Al 2s
Al 2p
0.0
5.0x10^4
1.0x10^5
1.5x10^5
2.0x10^5
2.5x10^5
3.0x10^5
3.5x10^5
1200 1100 1000 900 800 700 600 500 400 300 200 100
(c)
PAW 3.1
Counts /s
Binding energy (eV)
O 1s
C KLL
O KLL
O loss
C 1s
Al 2s
Al 2p
0.0
5.0x10^4
1.0x10^5
1.5x10^5
2.0x10^5
2.5x10^5
3.0x10^5
3.5x10^5
1200 1100 1000 900 800 700 600 500 400 300 200 100

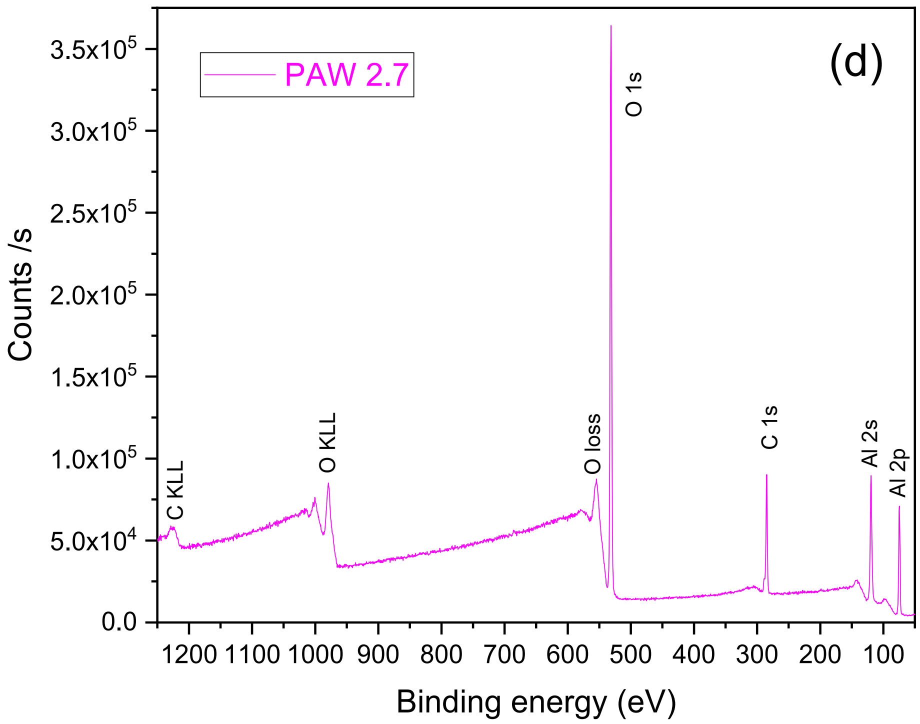

Figure S4. Survey XPS spectra for Al_2O_3 thin films deposited using: (a) DI water, (b) PAW at pH 3.5, (c) PAW at pH 3.1, and (d) PAW at pH 2.7.

The stoichiometry and carbon content of the film were determined through a high-resolution analysis of Al 2p (Fig. S5), O 1s (Fig. S6), and C 1s (Fig. S7) peaks.

The O/Al ratio was calculated by considering the area under the peaks corresponding to Al 2p and O 1s, which can be further determined using the equation provided below [31]:

$$\frac{O}{Al} = \frac{(Area\ O_O)/S_O}{(Area\ Al)/S_{Al}} \tag{S5}$$

In the equation (S5), S_o (0.71) and S_{Al} (0.23) represent the atomic sensitivity factors for oxygen and aluminum, respectively. To account for the stoichiometry of total oxygen species in the Al_2O_3 film, O_t/Al is calculated, where O_t is the sum of oxygen species (O_o) bonded with aluminum and chemisorbed oxygen species (O_c) with OH groups [31,32]. To determine the carbon content within the bulk of Al_2O_3 films, the C 1s peak was deconvoluted into three distinct peaks.

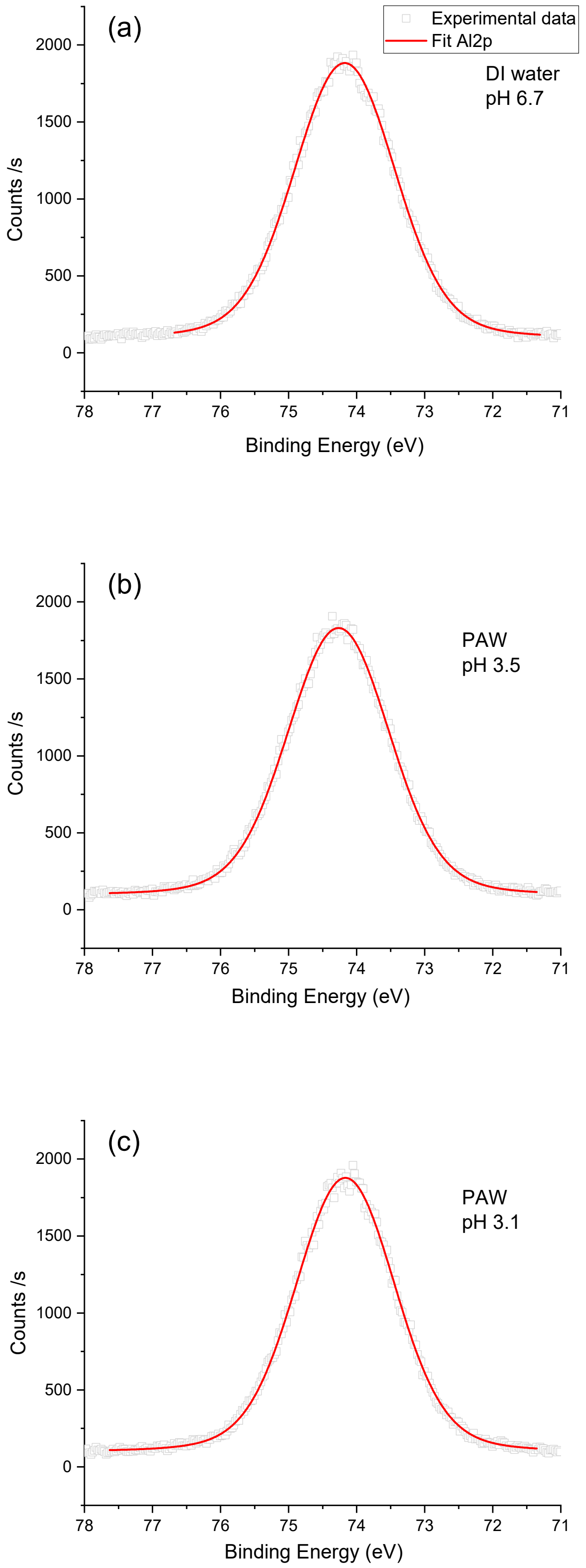
(a)
Experimental data
Fit Al2p
DI water
pH 6.7
Counts /s
Binding Energy (eV)
(b)
PAW
pH 3.5
Counts /s
Binding Energy (eV)
(c)
PAW
pH 3.1
Counts /s
Binding Energy (eV)

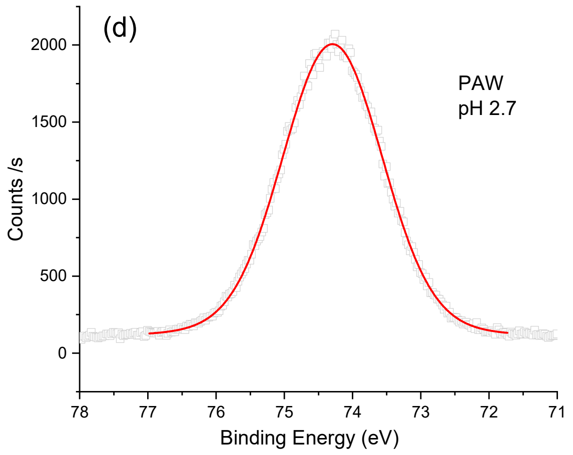

Figure S5. High-resolution XPS spectra of the Al2p peak and the corresponding deconvoluted peak for Al_2O_3 samples deposited using: (a) DI water, (b) PAW at pH 3.5, (c) PAW at pH 3.1, and (d) PAW at pH 2.7.

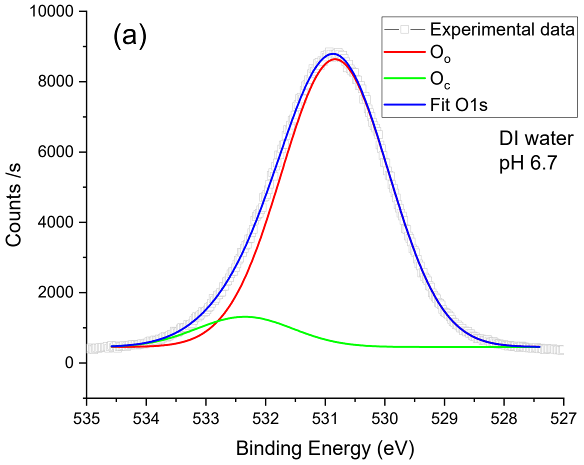

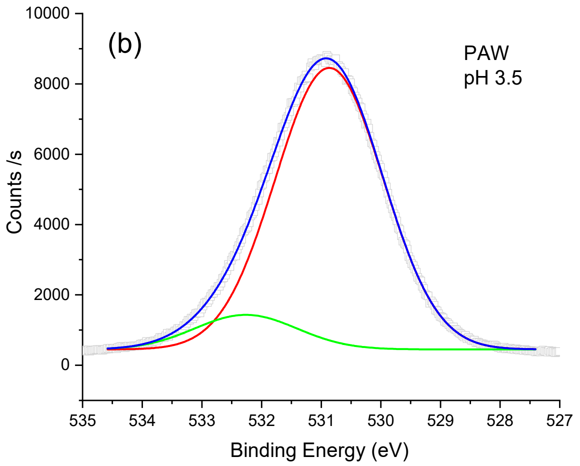

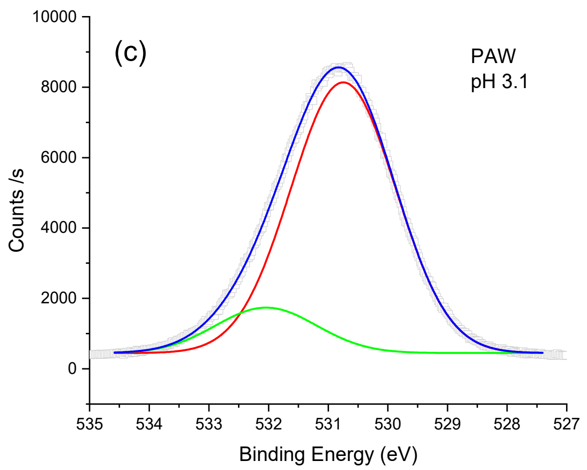

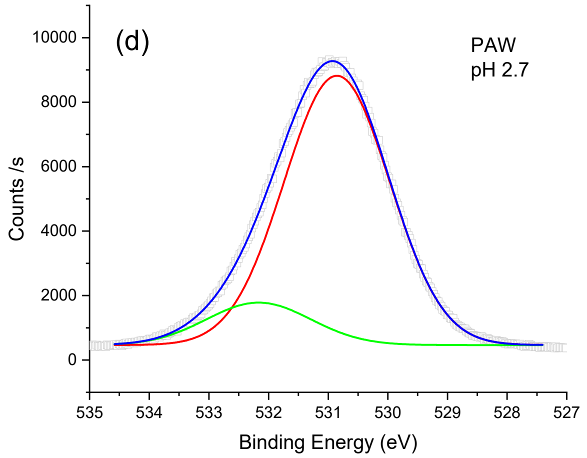

Figure S6. High-resolution XPS spectra of the O1s peak and the corresponding deconvoluted peaks for Al_2O_3 samples deposited using: (a) DI water, (b) PAW at pH 3.5, (c) PAW at pH 3.1, and (d) PAW at pH 2.7.

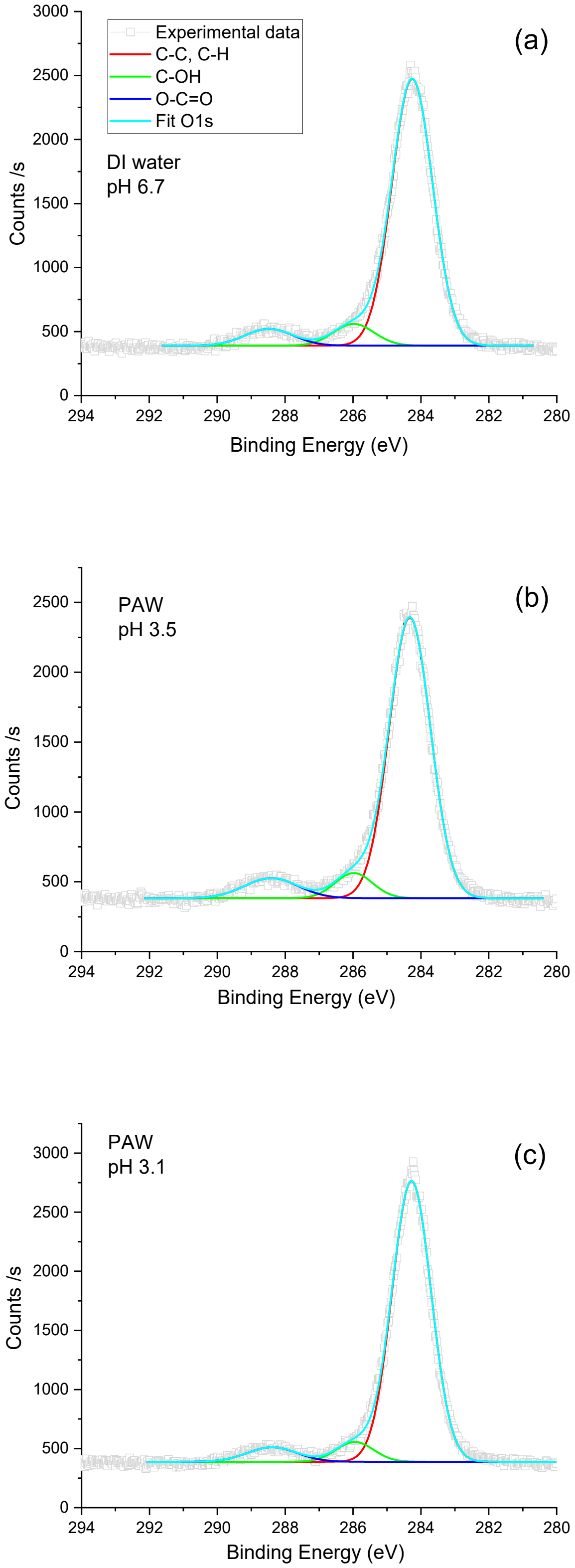
Experimental data
C-C, C-H
C-OH
O-C=O
Fit O1s
(a)
DI water
pH 6.7
Counts /s
Binding Energy (eV)
(b)
PAW
pH 3.5
Counts /s
Binding Energy (eV)
(c)
PAW
pH 3.1
Counts /s
Binding Energy (eV)

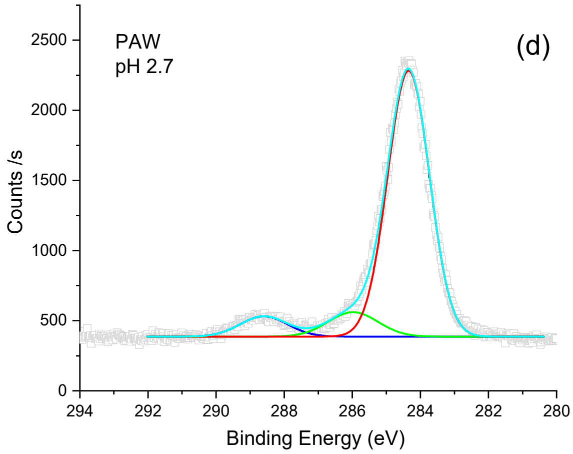

Figure S7. High-resolution XPS spectra of the C1s peaks and the corresponding deconvoluted peaks for Al_2O_3 samples deposited using: (a) DI water, (b) PAW at pH 3.5, (c) PAW at pH 3.1, and (d) PAW at pH 2.7.

The peak corresponding to the lowest binding energy of approximately 285 eV indicates surface-adsorbed C–C carbon, while the peaks at higher binding energies correspond to carbon species present in the bulk, which can be attributed to C–O–C (around 287 eV) and O–C=O (around 290 eV) [31]. The carbon content of the film (C %) was calculated using the following equation [31]:

$$C\ \% = \frac{(Area\ C_t)/S_C}{[\left(\frac{Area\ Al}{S_{Al}}\right)+\left(\frac{Area\ O_t}{S_O}\right)+\left(\frac{Area\ C_t}{S_C}\right)} \quad \text{(S6)}$$

where Area C_t represents the area under the peaks corresponding to carbon species present in the bulk, namely C–O–C and O–C=O. S_c (0.30) denotes the atomic sensitivity factor for carbon.

Finally, we adopted the procedure outlined by Iatsunskyi et al. [33] and Castillo-Saenz et al. [23] to calculate the energy band gap (E_g) of Al_2O_3 thin films. This method is based on the principle that the energy gap (E_g) corresponds to the difference in energy between the elastic peak (such as the oxygen peak, E_{O1s}) and the onset of inelastic losses (E_{loss}). To determine E_g, a linear fit is first applied to the loss spectra curve near the elastic peak. Then, the Shirley background fitting, which represents the "zero" background level, is subtracted. The point where the linear-fit line intersects with the background "zero" level marks the onset of inelastic losses. Consequently, the band gap energy is calculated as the difference between the elastic peak energy and the onset of inelastic losses, given by $E_g = E_{loss} - E_{O1s}$.
